# Costs and benefits of bevacizumab vial sharing for the treatment of retinal diseases

**DOI:** 10.1186/s12889-019-7562-y

**Published:** 2019-09-11

**Authors:** Sávio Lima Sodré, Italo Antunes França Barbosa, Israel Emiliano Pacheco, Felipe de Queiroz Tavares Ferreira, Milton Agrizzi David, Mauricio Abujamra Nascimento, Carlos Eduardo Leite Arieta, Jose Paulo Cabral de Vasconcellos

**Affiliations:** Universidade Estadual De Campinas (UNICAMP), Cidade Universitária Zeferino Vaz - Barão Geraldo, Campinas, SP 13083-970 Brasil

**Keywords:** Anti-vascular endothelial growth factor, Bevacizumab, Repackaging, Vial sharing, Public hospitals, Brazil

## Abstract

**Background:**

Antiangiogenic therapy has proved to be an important therapeutic tool for many retinal vascular diseases; however, its availability is limited in developing countries. This study sought to describe the bevacizumab vial sharing process and to evaluate the impact of this repackaging system on the costs incurred in a Brazilian public hospital.

**Method:**

This retrospective study compared the number and costs of intravitreal antiangiogenic injections approved via court order in the first year of the study (2015) to the number and costs of the bevacizumab injections provided through the use of vial sharing in the second year of the study (2016).

Vial sharing consists of the traditional process used to repackage bevacizumab; in this case, however, the drug samples used were the residual volume from the preparation of bevacizumab for oncology patients. The hospital adhered to the guidelines established by the Brazilian Health Surveillance Agency (ANVISA).

**Results:**

In the first year of the study and using medication obtained through court orders, 550 intravitreal injections were performed in the ophthalmology ambulatory care center. Based on local pricing tables, the total cost of the medication was BRL$1,036,056.25 (USD$267,546.58), and the average cost of each application was BRL$1883.74 (USD$486.45).

In the second year of the study, 1081 intravitreal applications were performed at the same hospital using doses obtained through bevacizumab vial sharing. The total cost was BRL$21,942.49 (USD$5663.30) and the per-unit cost was BRL$20.30, or USD$5.23 (a savings of 97.88%).

**Conclusion:**

This study found that bevacizumab vial sharing led to a significant reduction in public health care costs associated with antiangiogenic treatment and increased the availability of the drug to public health care patients. These results can be extrapolated to other types of drugs and health care systems.

## Background

Since being introduced on the market, anti-vascular endothelial growth factor (anti-VEGF) drugs, also known as antiangiogenics, have become widely used as the treatment of choice for retinal diseases such as diabetic macular edema and wet age-related macular degeneration (wet ARMD), both of which are frequent causes of blindness in many populations [1, 2]. In Brazil, three antiangiogenics are available for the treatment of retinal vascular diseases: ranibizumab (Lucentis®), aflibercept (Eylia®), and bevacizumab (Avastin®), the last of which is used off label in ophthalmology [3].

In Latin America, there are many obstacles to obtaining anti-VEGF therapy. These barriers include the high cost of care, the refusal by both private and public health care providers to cover these medications, and patients’ limited access to retina specialists [4]. According to 2013 data from the Brazilian Institute of Geography and Statistics (IBGE), 27.9% of the Brazilian population is covered by a private health care plan; the vast majority of the population relies on the Brazilian National Public Health Care System (SUS) for medical care [5]. Even in regions where treatment from SUS or private health care plans is available, access to medication is hindered by bureaucracy. Patients—particularly those with limited mobility or without access to reliable transportation—tend to give up on trying to obtain anti-VEGF therapy or, when they are finally able to receive care, are no longer within their treatment window. This situation reflects the importance of improving access to effective interventions [4].

Bevacizumab is a humanized monoclonal anti-VEGF antibody that was originally developed and approved to treat colorectal cancer [6]. It has not been approved for intravitreal use by Brazilian health agencies; however, after careful analysis of the evidence available, which has demonstrated its safety and efficacy at rates comparable to those of other anti-VEGF agents [7–12], the Brazilian Ministry of Health issued an official opinion recommending this use [13–15].

In addition to the similarities between bevacizumab and the other anti-VEGF drugs in their efficacy against retinal diseases, an important advantage is the lower cost of the former medication [1–3, 7]. The per-patient cost of bevacizumab may be four to forty times lower, depending on the dosages in which bevacizumab is repackaged [8–12]. The cost-effectiveness provided to patients has also been proven to be higher than that provided by ranibizumab or aflibercept [12]. Bevacizumab has been repackaged for intravitreal injection by compounding the drug into vials for multiple patients in an attempt to reduce the cost of treatment while complying with the guidelines established by the Brazilian Health Surveillance Agency (ANVISA) [16].

Despite efforts in the field of oncology to reduce the amount of leftover medication, waste cannot be completely avoided due to the fact that bevacizumab doses depend on patient weight [6]. In the case of bevacizumab, vial sharing consists of the same compounding process outlined in Brazil’s ANVISA guidelines [16], though with the use of quantities of the drug left over from compounding processes performed on bevacizumab doses destined for oncology patients. These leftover quantities are repackaged for intravitreal use in an attempt to reduce costs, increase the availability of antiangiogenics for ophthalmologic treatment, and provide patients with the same level of care while reducing waste in the public health care system. The benefits found in this study reflect the importance of health care policies that support the repackaging of medications and encourage the least wasteful use of public resources.

## Methods

### Objective

To calculate the reduction in costs per patient treated with intravitreal anti-VEGF and to demonstrate a bevacizumab repackaging model as a viable and more effective option for public health care systems in the treatment of retinal diseases such as diabetic macular edema, ARMD, and retinal vein occlusions. To compare the direct costs of antiangiogenics used in the ophthalmology ambulatory care center of the Clinical Hospital of the State University of Campinas (UNICAMP) before the implementation of this shared repackaging system to the costs to the department after its introduction.

### Study design

This was a technical, observational, and retrospective study performed in the Retinal Ambulatory Care Center of the Clinical Hospital of the State University of Campinas (HC-UNICAMP). This hospital is part of Brazil’s public health care system (SUS), in which many patients must obtain a court order to receive anti-VEGF intravitreal injection treatment.

### Study subjects

The subjects included in this study were patients from the Retinal Ambulatory Care Center of HC-UNICAMP who received the intravitreal injection of an anti-VEGF drug between August 2015 and July 2017 due to their low visual acuity associated with macular abnormalities or thickness as determined by both macular optical coherence tomography (OCT) and an ophthalmologic examination, and secondary to at least one retinal disease (diabetic macular edema, macular edema secondary to retinal vein occlusion, or wet ARMD). These patients were transferred from regional health care departments or sought care from HC-UNICAMP on their own accord. They were selected because they represented the population that depends exclusively on public health care services in the city of Campinas.

The perspective used in this study is classified as one of a third-party payer system, which represents the Brazilian National Public Health Care System (SUS); in this system, costs associated with factors such as missed days of work and transportation are not included and are the patient’s responsibility [17].

The inclusion criteria in this study are consistent with the criteria used in the protocol at the study institution for indicating treatment with anti-VEGF drugs. The criteria for each specific vascular condition are as follows:

### Wet ARMD

- Active neovascular membrane on the retina (evaluated using fundus biomicroscopy, OCT, and angiography with fluorescein);
- No fibrous components in or around the lesion;
- No atrophy of the external retinal layers in or around the lesion as determined by OCT;
- Corrected visual acuity between 20/40 and 20/400 as per the ETDRS chart and justified by wet ARMD;

### Macular edema secondary to retinal vein occlusion

- Presence of intraretinal cysts or subretinal fluid in the macular area;
- Central thickness ≥ 250 μm;
- External retinal layers intact;
- Corrected visual acuity between 20/30 and 20/200 as per the ETDRS chart;
- Less than 1 year of progression/development;

### Diabetic macular edema

- Diabetic macular edema with central involvement
- Corrected visual acuity < 20/30 as per the ETDRS chart and due to EMD
- foveal thickness > 250 μm as per OCT

### Study procedure

Patient information was obtained through data collection from patient medical records and appointments at the local retinal ambulatory care center for the intravitreal application of an anti-VEGF agent between August 2015 and July 2017. An analytical scope of 2 years was selected because it provided 1 year of traditional treatment that could be compared to 1 year of treatment provided after the study intervention and because it reflected sufficient time for a comparative analysis.

To better analyze the differences between the patients treated in the 2 years of the study in terms of costs and availability of the intravitreal antiangiogenic injections, this study evaluated the number of patients, the medications used (bevacizumab, ranibizumab, and aflibercept), and the costs associated with intravitreal injections approved via court order between August 2015 and July 2016 (the first year of the study). This study also compared the number of patients treated with intravitreal injections and the costs of bevacizumab in cases of vial sharing, which was implemented from August 2016 to July 2017 (the second year of the study).

Because the prices were provided retrospectively, they come from different sources and years; neither source considered prices for longer than a one-year period, and because the long-term benefits associated with this treatment were not considered herein, the concept of a discount rate is not relevant to this study [17].

The study relied on primary research; the data collection process is described below and generated its own set of quantitative information. The reference cost of each medication obtained via court order in the first year of the study was based on the pricing tables for drugs sold to the Brazilian government (known locally as the PMVG) as published by the Brazilian Department of Drug Market Regulations (CMED) [18]. The retail price of bevacizumab used in the second year of the study, during which time the medication was obtained directly from the hospital in question, was provided by study site administrators. Because the study drug would have been discarded, there was no opportunity cost associated with the use of the drug; however, the costs of the professionals and physical space involved in the repackaging process have been included.

The data obtained were organized in an Excel spreadsheet and descriptively analyzed. The exchange rate used for the Brazilian real (BRL) to the US dollar (USD) was the average exchange rate of the dollar in July 2018 (3.8745 Brazilian reais to the US dollar) [19].

### Ethical considerations

The study was approved by the local Research Ethics Committee CEP (Comitê de Ética em Pesquisa) under committee registry number CAAE (Certificado de Apresentação para Apreciação Ética) 60,695,516.1.0000.5404. This study received no outside funding and has no conflicts of interest. This study received assistance in the form of information provided by the oncology pharmacy of HC-UNICAMP, which served as a non-monetary form of support.

Due to its classification as retrospective and observational, this study was not required to follow the guidelines of a population study, or to obtain informed consent forms from patients.

### Bevacizumab repackaging

In health care centers, repackaging (or compounding) is a procedure led by pharmacists. It consists of removing a given infused drug from its original packaging and dividing it into smaller doses in smaller vials in a way that maintains its quality and identifying information. In the hospital pharmacy in this study, bevacizumab is repackaged in a class II B2 biological safety cabinet according to guidelines established by ANVISA [16].

ANVISA has also established rules and regulations regarding the compounding, storage, transportation, and distribution of medications. These rules and regulations also outline minimum prerequisites for a given institution to perform these processes. The prerequisites, with which the local hospital complies, outline the need for common areas, a room for cleaning and sterilizing products, a weighing area, a cleanroom exclusively for compounding and container closure, a review area, an area for quarantine, labeling, and packaging, and the use of exclusive locker rooms (changing rooms) by employees. As per these guidelines, the clean room used to compound and close sterile preparations must be separate and used exclusively for this purpose. It must contain air filters to retain particles and microorganisms and to guarantee recommended air quality as per ISO Class 5 (100 particles / ft^3^ of air) or a biological safety cabinet in accordance with ISO Class 5; it must be in an area consistent with ISO Class 7, and it must exhibit positive pressure [16].

Vial sharing consists of this same process used to repackage bevacizumab; in this study, the quantities of the drug used were those that were left over from the preparation of bevacizumab for oncology patients. This excess volume was aspirated and repackaged in a biological safety cabinet. The product was labeled and stored at adequate temperatures levels (between 2 °C and 8 °C) to ensure the safe transportation of the medication to the ophthalmology ambulatory care center where the intravitreal injection was performed with proper antiseptic care prior to injection. The vial sharing process was performed using ultrafine (0.03 ml) syringe-needle combinations with no dead volume. With 1 ml of the drug, approximately 20 doses for intravitreal use (1.25 mg / 0.05 ml per patient) were produced per week.

## Results

In the first year of the study and using medication obtained through court orders, 550 intravitreal injections were performed in the ophthalmology ambulatory care center of the study site (192 of which were bevacizumab, 347 of which were ranibizumab, and 11 of which were aflibercept). Based on local pricing tables [18], the total cost of the medication was BRL$1,036,056.25 (USD$267,546.58), and the average cost of each application was BRL$1883.74 (USD$486.45). Details are presented in Table 1.

**Table 1:** Per-unit and total cost^a^ per medication according to government from August 2015 to June 2016

MEDICATION	NUMBER OF APPLICATIONS	COST (in BRL$/USD$)	TOTAL COST (in BRL$/USD$)
Bevacizumab (4 ml)	192	BRL$1291.84 USD$333.42	BRL$248,033.28 USD$64,016.84
Ranibizumab	347	BRL$2176.72 USD$561.80	BRL$755,321.84 USD$194,946.92
Aflibercept	11	BRL$2972.83 USD$767.28	BRL$32,701.13 USD$8440.09
TOTAL	550		BRL$1,036,056.25 USD$267,455.47

^a^Maximum retail prices of drugs sold to the Brazilian government (known locally as the PMVG)

In the second year of the study, 1081 intravitreal applications were performed at the same hospital using doses obtained through bevacizumab vial sharing, with an isolated cost of BRL$17,455.98 (USD$4514.28). Given the fact that approximately 1 hour is required to repackage 20 doses (and that doses are administered weekly), the additional cost for the specialized medical professional must be included (BRL$38.47 as reported in the wage table for the study institution [20]), as well as the per-hour cost of reserving the cleanroom (approximately BRL$55.00 as charged by the UNICAMP Department of Biomedical Engineering). Therefore, the total cost of vial sharing in the second year of the study was BRL$21,942.49 (USD$5663.30), and the per-unit cost was BRL$20.30 (USD$5.23).

The comparison between the first and second year revealed a 97.88% reduction (BRL$1,014,113.26, or USD$261,740.55) in the total cost of a year of injections, as well as a 96.54% increase in the number of injections performed after bevacizumab vial sharing was implemented. The analysis of the savings from the use of bevacizumab rather than the currently approved antiangiogenics if the treatment had been carried out in the 2 years of the study is shown in Table 2.

**Table 2:** Savings if the treatment had been carried out in the 2 years of the study

		1631 BEVACIZUMAB VIAL SHARING	COST DIFFERENCE
*1631 Bevacizumab PMVG*	BRL$2,106,991.04 USD$543,808.02	BRL$33,109.30 USD$8545.43	BRL$2,073,881.74 USD$535,264.35
*1631 Ranibizumab PMVG*	BRL$3,550,230.32 USD$916,295.80	BRL$33,109.30 USD$8545.43	BRL$3,517,121,02 USD$907,761.26
*1631 Aflibercept PMVG*	BRL$4,848,685.73 USD$1,251,433.68	BRL$33,109.30 USD$8545.43	BRL$4,815,576.43 USD$1,242,889.77

If repackaged bevacizumab doses had been used in the 550 injections in the first year, the cost for the year would have been BRL$11,165.00, or USD$2881.66 (a savings of BRL$1,014,113.76, or USD$261,740.55). Similarly, if the injections approved via court order had been continued at the same rate during the second year, the total cost to treat the patients reached in the second year would have been BRL$2,036,322.94 (USD$525,570.51).

However, given the fact that this study was performed using vial sharing and small aliquots of bevacizumab that the hospital would have otherwise been discarded, the cost of the medication for the ophthalmology department was nil and the savings achieved using this method were even more pronounced.

## Discussion

As this study shows, the reduction in cost through the use of repackaging allowed for a public teaching hospital to increase the number of intravitreal injections available despite the limited resources provided to the Brazilian public health care system.

In the first year of the study, 550 anti-VEGF injections were applied at the study site after patients obtained court orders to receive treatment from August 2015 to July 2016. The total cost was BRL$1,036,056.25 (USD$267,546.58). Because bevacizumab doses obtained through vial sharing for this purpose cost BRL$20.30 (USD$5.23) after considering the added cost of the physical space and personnel required for the process, the equivalent cost to treat these same 550 patients would have been BRL$11,165.00, or USD$2881.66 if vial sharing had been implemented or if smaller original doses had been available. In this case, this difference represents a 98.92% savings (BRL$1,024,891.25 or USD$264,522.19).

Bevacizumab vial sharing was implemented at the study site in the second year of the study, and 1081 intravitreal injections were performed in from August 2016 to July 2017. These treatments represent a 96.54% increase in the number of applications relative to the year prior, when vial sharing had not been implemented. These numbers provide evidence that, at this hospital, the main factor limiting the number of applications in the first year was the availability of the drug. Even with the crucial participation of the hospital pharmacy department in the introduction of the repackaging system, there was no need for structural or personnel changes for its implementation.

Though antiangiogenic drugs are not considered equivalent, if an ophthalmology department within Brazil’s public health care system provides only one high-cost medication free of charge to patients, patients that do not respond to that specific treatment are required to request different medications through court order. As of mid-2016, however, Brazil found itself at the peak of one of its worst financial crises in its history. For this reason, approval processes of court orders for high-cost medications became even more drawn out, and approvals were not certain. The extreme scarcity of government-provided antiangiogenic drugs was the main reason that this new method was created, since physicians needed to provide their patients with these high-cost medications and public hospitals wanted to make good use of the limited supply. Because repackaged bevacizumab became the only way to make anti-VEGF drugs available at this institution, only this treatment was used in the second year of the study. In the second year, cases of refractory cystoid macular edema, for example, were treated with triamcinolone or macular grid laser photocoagulation (alternative therapies available in the department). In any case, only anti-VEGF drug therapy was considered in this study.

The cost of bevacizumab was maintained in the calculations in the second year of this study: though the doses used were obtained from excess quantities of the drug that would have otherwise been destroyed, the acquisition of the drug produced administrative costs for the State Health Secretariat. In light of this situation, it can also be argued that the cost of the drug became negligible for the ophthalmology department.

This situation also reflects one of the most important points of this study: if vial sharing processes such as these are implemented, anti-VEGF treatment can become available in hospitals and health care centers where even traditional repackaging is not financially viable. These results therefore highlight the fact that vial sharing procedures for high-cost medications represent a useful option for centers facing financial constraints.

It is also important to note that, by providing compounded doses of the medication, the study site was able to reduce the amount of time between indication and the start of treatment, in addition to avoiding the administrative costs associated with court orders.

High-cost medication waste is not only a problem in Brazil. Even when hospitals and health care systems prioritize efforts to avoid wasting infused drugs, wasted volumes may vary between 1 and 33%. Bevacizumab waste in the United States in 2016 was estimated at 9% [21].

Chen et al. analyzed the sterility, stability, and efficacy of bevacizumab repackaged into multiple doses. Their results demonstrate that, if proper antiseptic precautions are taken during the manipulation and use of this medication (and if the drug is stored at 4 °C for no more than 6 months), compounded doses of bevacizumab will remain sterile and anti-VEGF activity will remain minimally (less than 10%) degraded during this period [22].

Another challenge to the implementation of this medication is the approval of bevacizumab for ophthalmologic pathologies in Brazil. Despite the Ministry of Health’s interim approval of bevacizumab for ophthalmologic use in the country in 2016 [13], the ROCHE® laboratory issued an official statement to report that it does not agree with the temporary authorization of the use of the drug for off-label treatment in eyes. The company believes that such off-label usage should be the sole responsibility of patients and professionals who accept the potential risks that have been associated with this treatment procedure [23]**.** This corporate position limits the ophthalmological use of this medication to institutions that, like this one, perform scientific studies on the drug. Patients must be informed of the situation and must sign consent forms acknowledging the experimental nature of the treatment and potential risks of using this medication in their eyes.

In Brazil, the resources allocated to public health are insufficient and poorly managed. Brazil is a developing country that has experienced both demographic transitions and economic crises in recent years, both of which have had substantial impacts on public health [24]. The resources available must therefore be used economically, particularly in cases of high-cost medications [25]. A compounding factor in this situation is that ARMD is considered the main cause of legal blindness among those over 50 years of age in some industrialized nations [26, 27]. Furthermore, researchers estimate that the global prevalence of diabetes mellitus will increase from 2.8% (171 million) as of the year 2000 to 4.4% (366 million) by 2030, with the most significant increases in developing countries [28–30].

According to the Brazilian National Committee for Health Technology Incorporation (CONITEC), the budgetary impact for treating wet ARMD in the general population in the country in 5 years will be BRL$300,949,504.64 (USD$77,674,410.80) in the case of ranibizumab and BRL$50,236,922.63 (USD$12,966,040.17) in the case of bevacizumab (considering repackaging into four doses from each 0.3 ml vial of ranibizumab and 40 doses from each 4 ml vial of bevacizumab) [14].

In the case of treatment for diabetic macular edema, CONITEC has also estimated that the use of repackaging in this situation would result in a three-year budgetary impact ranging from BRL$143,002,198.00 or USD$36,908,555.43 (if repackaged bevacizumab were used) to BRL$12,359,563,100.00 or USD$3,189,976,280.81 (if single-dose vials of ranibizumab were used) [15].

Assuming the difference in cost identified by CONITEC for the use of repackaged bevacizumab rather than ranibizumab (four doses in the treatment of ARMD or a single dose in the treatment of diabetic macular edema), if repackaged bevacizumab were used in the Brazilian public health care system at the repackaged dose value found in this study (BRL$20.30 or USD$5.23), the government would be able to provide 12,350,376.49 doses for the treatment of wet ARMD at 5 years and 601,801,029.65 doses for diabetic macular edema at 3 years.

It is important to note that CONITEC does not consider the costs of the repackaging process in its assessment of the economic impact of repackaging bevacizumab using the methods performed in this study. The cost of vial sharing may be financially viable due to the substantial savings provided by the use of this drug in this way.

The cost of the syringe is also negligible relative to the possible savings provided by the use of this medication, in part because the syringes used in the repackaging process are also used in the application of the drug. The cost of follow-up care was not considered, since this care is provided regardless of the medication used.

The Consolidated Health Economic Evaluation Reporting Standards (CHEERS) statement was developed by a task force as part of a broader initiative to facilitate and encourage economic assessments in health care; however, because the current study included a simple economic analysis without a consequence analysis or a consideration of health-related outcomes, some items of the CHEERS protocol do not apply to this study [31].

Other limitations of our study are its retrospective nature, the need for a specifically structured hospital pharmacy, and the training required for pharmaceutical professionals in order for the hospital to comply with the rules of the local health surveillance agency.

## Conclusion

This study provides a model for the repackaging of bevacizumab through a vial sharing process as a viable option for public hospitals. In Brazil, this process complies with Brazilian Health Surveillance Agency (ANVISA) regulations and has been found to increase availability and reduce the cost of treatment for diseases such as diabetic macular edema, age-related macular degeneration, and retinal vein occlusions. In the comparison between the first year of the study (when treatment was available to patients only through a court order) and the second year of the study (when repackaging via vial sharing was implemented), the use of compounded doses of bevacizumab resulted in a 97.88% savings on drug costs and a 96.54% increase in the availability of treatment, as well as a reduction in wasted aliquots.

The increased number of patients treated and the reduction in costs represent the benefits provided to the public health care system and patients alike. Due to the promising results of this study, further research and analysis are encouraged to evaluate the effects of repackaging of high-cost medications and of other measures on the efficient use of public resources.

## Data Availability

The datasets generated and analyzed during the current study are not publicly available; the information is only part of physical personal medical records; this information is available from the corresponding author upon reasonable request.

## Abbreviations

ANVISA: The Brazilian Health Surveillance Agency (Agência Nacional de Vigilância Sanitária)
ARMD: Age-related macular degeneration
BRL: Brazilian reais
CAAE: Certificado de Apresentação para Apreciação Ética - Certificate of presentation for ethical consideration – an ethics evaluation certificate
CEP: Comitê de Ética em Pesquisa - Research Ethics Committee
CHEERS: Consolidated Health Economic Evaluation Reporting Standards
CMED: Brazilian Department of Drug Market Regulations (Câmara de Regulação do Mercado de Medicamentos)
CONITEC: Brazilian National Committee for Health Technology Incorporation (Comissão Nacional de Incorporação de Tecnologias no SUS)
HC-UNICAMP: Clinical Hospital of the State University of Campinas (Hospital das Clínicas da Universidade Estadual de Campinas)
ISO: International Organization for Standardization
OCT: Optical coherence tomography
PMVG: Maximum Prices for Sales to the Government – the pricing tables for drugs sold to the Brazilian government (Preço Máximo de Venda ao Governo)
SUS: The Brazilian National Public Health Care System (Sistema Unico de Saúde)
UNICAMP: State University of Campinas (Universidade Estadual de Campinas)
USD: United States Dollar
VEGF: Vascular endothelial growth factor
